# Delays in extubation following elective adult cardiac surgery

**DOI:** 10.1186/cc13375

**Published:** 2014-03-17

**Authors:** J Parmar, J Clarke, G Lau, R Porter, C Allsager

**Affiliations:** 1 Glenfield Hospital, University Hospitals of Leicester, UK

## Introduction

Early extubation post coronary artery bypass grafting does not increase perioperative morbidity and reduces the length of stay (LOS) in the ICU and in hospital [1]. Use of low-dose opioid- based general anaesthesia and time-directed protocols for fast- track interventions does not increase mortality or postoperative complications in low-moderate-risk patients and has been found to have a reduced time to extubation and shortened ICU stay [2]. Our mean time to extubation is 6 hours, although patients are assessed to be safe to be weaned from mechanical ventilation at 2 hours following arrival in the ICU. This study aims to identify factors that delay extubation in patients undergoing routine cardiac surgery at our institution.

## Methods

A prospective analysis was performed on all patients post adult cardiac surgery from 14 May 2013 to 10 July 2013. Emergency surgical patients and those with intraoperative complications were excluded.

## Results

A two-sample *t *test was used to analyse the data. Patient demographics are presented in Table 1. There were significant delays in time of extubation in those who received morphine prior to extubation compared with those that did not (*P *= 0.0184) (Table 2). There were no significant differences in LOS in ICU or hospital. Factors such as age, EUROSCORE and type of operation did not have an influence on time to extubation.

**Table 1:** Patient demographics

Age (years)	66 (10.1)
EUROSCORE (%)	2.90 (1.84)

**Table 2:** Morphine versus no morphine

	Morphine	No morphine
Patient number	51	20
Time to extubation (hours)	7:48 (4:42)	4:48 (2:19)
LOS ICU (hours)	51:21 (55:16)	40:06 (28:47)
LOS hospital (days)	9.83 (5.01)	10.8 (13.5)

## Conclusion

Administering morphine prior to extubation causes significant delays in weaning from mechanical ventilation. We plan to introduce intraoperative and postoperative protocols to facilitate rapid weaning from mechanical ventilation for elective cardiac surgical patients.
